# Morphological Patterns of Sarcoidosis and Clinical Outcome: Retrospective Analysis through a Multidisciplinary Approach

**DOI:** 10.3390/diagnostics10040212

**Published:** 2020-04-11

**Authors:** Giulio Distefano, Ada Vancheri, Monica Palermo, Francesco Tiralongo, Pietro Valerio Foti, Letizia Antonella Mauro, Carlo Vancheri, Antonio Basile, Stefano Palmucci

**Affiliations:** 1 Radiology Unit 1, Department of Medical Surgical Sciences and Advanced Technologies “GF Ingrassia”–University Hospital “Policlinico-Vittorio Emanuele”, University of Catania, 95123 Catania, Italy; monica.palermo91@gmail.com (M.P.); tiralongofrancesco91@hotmail.it (F.T.); pietrofoti@hotmail.com (P.V.F.); mauroletizia@tiscali.it (L.A.M.); basile.antonello73@gmail.com (A.B.); spalmucci@sirm.org (S.P.); 2 Department of Clinical and Experimental Medicine, University of Catania, Regional Referral Centre for Rare Lung Disease, 95123 Catania, Italy; adact1@hotmail.it (A.V.); vancheri@unict.it (C.V.)

**Keywords:** sarcoidosis, high-resolution computed tomography (HRCT), pulmonary function test (PFT), atypical pattern, computer tomography activity score (CTAS), score, chest, pulmonary, interstitial lung diseases

## Abstract

The aim of this work was to verify the correlations between different pulmonary morphological patterns and functional outcomes in sarcoidosis patients, using a validated score for the comparison between the high-resolution computed tomography (HRCT) of patients belonging to different imaging patterns. From the electronic database of the reference center for interstitial lung diseases of our University Hospital, we retrospectively selected 55 patients with a diagnosis of sarcoidosis according to the American Thoracic Society (ATS) criteria; we evaluated the initial HRCT examination and pulmonary function tests collected at baseline and after a year. Patients were divided into typical (48% of patients) and atypical (52%) HRCT patterns, and a computer tomography activity score (CTAS) was associated with each HRCT appearance detected; clinical history, impact of therapy, and extra-thoracic locations were also considered. We found that worsening of diffusing capacity for carbon monoxide (DLCO) is related to the CTAS (*r* = −0.20, *p* = 0.01), and there was an inverse correlation between the variation of forced vital capacity (FVC) and the value of the CTAS (*r* = −0.30, *p* = 0.23) in the subgroup of patients with atypical patterns. CTAS were higher in patients with extra-pulmonary localizations (*p* = 0.05) and the subgroup of patients with extra-thoracic locations and atypical manifestations had a greater worsening in terms of variation of FVC (*p* = 0.03) and DLCO% (*p* = 0.04). No difference between treated and untreated patients was found.

## 1. Introduction

### 1.1. Background

Sarcoidosis is a systemic granulomatous pathology of unknown etiology, first described in 1899 by Boeck who found “epithelioid cells with a pale nucleus and some giant cells” in a benign skin lesion [1]. This disease is not considered rare among interstitial lung diseases (ILD), and some important epidemiological variations have been reported related to geographical and ethnic factors. The maximum incidence is registered in the northern European countries (up to 40 cases per 100,000 inhabitants), whereas the minimal incidence has been reported in the Eastern World (1:100,000 in Japan) [2]; it appears to be more common in African Americans than Whites [3]. The prevalence in the Italian population is estimated at 10:100,000, with incidence peaks among young adults and more frequently in women [4]. The etiology of sarcoidosis is probably multifactorial, where genetic and environmental factors play important roles; aberrant interaction of CD4^+^ T lymphocytes with antigen-presenting cells (APC) represents the initial pathogenic event. The development of granulomas represents the hallmark of this disease, whose clinical course is very variable and often self-limiting.

Pulmonary fibrosis is the most serious complication of the disease and occurs in up to 25% of cases [5]. The diagnosis of sarcoidosis is generally based on clinical and radiological features and on the demonstration of non-caseous granulomas on histological examination [6]; it has also been shown that bronchoalveolar lavage (BAL) alterations can be sufficient in patients with clinical and radiological suspicion [7]. Among the imaging modalities, high-resolution computed tomography (HRCT) has proven to be accurate in demonstrating parenchymal and mediastinal lesions due to sarcoidosis [8].

**Figure 1:**
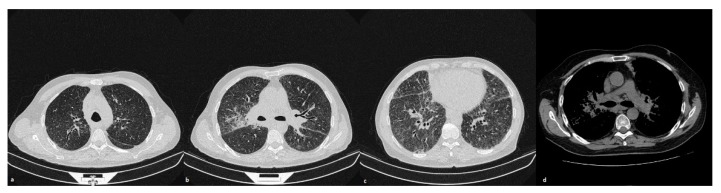
A patient affected by pulmonary sarcoidosis. Axial scan passing through the bases (**a**), through the origin of the pulmonary artery (**b**) and through the apices (**c**), lung windows settings: tiny nodules distributed in a random hiliary-like manner are shown; (**d**) using the mediastinum window settings it is possible to appreciate the presence of bilateral symmetric hilar lymphadenopathy.

The utilization of computed tomography (CT) scans is justified, in particular for patients with clinical suspicion of sarcoidosis and a negative X-ray, or in cases with atypical presentation forms; it is also useful for assessing complications and defining cases with advanced lung disease that cannot benefit from potentially toxic therapies [8]. In sarcoidosis, pulmonary involvement may occur with a variety of different patterns. A typical pattern is represented by the presence of lung nodules and symmetric bilateral mediastinal and hiliary lymphadenopathies (Figure 1); unilateral, isolated, and/or calcified nodes could be considered as an element of atypia.

Some fibrotic changes such as linear opacities, interlobular septa thickening and traction bronchiectasis are considered features of typical pattern. The presence of bubbles, cysts, honeycomb, emphysema, and intralobular septa thickening constitutes an atypical presentation. Ground-glass opacities (GGO) are considered as a typical feature by some authors [6], while others assign them to the atypical pattern classification [9]. Finally, confluent or mass-like opacity, halo sign, airway alterations, and mosaic-attenuation patterns are considered atypical [9]. It is unclear whether the radiological pattern has any relevance on outcome and therapy response, although a recent study suggests that persistence of the inflammatory picture is a stronger negative predictive index than the initial pattern [6]. The severity of sarcoidosis depends on several clinical and laboratory factors. In order to produce a reproducible assessment, an objective score was proposed by Wasfi et al. [10], which takes into account demographic, functional, and organ involvement parameters. As there is a correlation between the disease activity parameters and the extension of some characteristic lesions at HRCT (including nodules, ground-glass opacities, interlobular septa thickening, and consolidations), a new score, exclusively based on radiological findings, has been recently proposed. This score, called the computer tomography activity score (CTAS), allows us to stratify the patients in relation to the intrathoracic extension of the disease, and also allows us to compare different samples in relation to the severity of the condition and to the response to treatment [11,12].

### 1.2. Aim

The aim of this study is to verify the correlations between different pulmonary morphological patterns and the clinical and functional outcomes in patients affected by sarcoidosis.

## 2. Materials and Methods

### 2.1. Study Design, Setting, and Participants

We selected patients who were assessed at our Regional Centre for Interstitial and Rare Lung Disease from January 2015 to April 2019 for pulmonary sarcoidosis. For each patient, we retrospectively evaluated clinical history, functional respiratory tests, and imaging data.

The following eligibility criteria were adopted: patients with pulmonary sarcoidosis diagnosed according to the American Thoracic Society / European Respiratory Society / World Association for Sarcoidosis and Other Granulomatous Disorders (ATS/ERS/WASOG) criteria [4], the availability of a complete clinical history, at last one-year clinical follow up, at least two HRCT scans during an active phase of the disease, at least two complete pulmonary function test (PFT), and a six-minute walking test (6MWT). We excluded patients with active neoplastic disease, history of chronic infections (viral, bacterial, or fungal), patients with proven or suspected rheumatologic diseases, and those with advanced pulmonary fibrosis.

### 2.2. Protocol Details

Since not all patients performed the imaging tests in our Radiology Institute, we only considered chest CT scans that were compatible with HRCT standard protocol [13], according the following technical parameters: caudo-cranial monophasic scan in inspiratory apnea, thin-section CT images ranged between 0.625 mm and 1.5 mm, sharp kernel imaging reconstruction, contiguous or overlap images, and no contrast media administration. Radiological exams were evaluated in consensus by two radiologists with proven experience in pulmonary interstitial pathology, blinded to clinical or functional data. Parenchymal alterations were distinguished according to criteria of typicality and atypicality (Table 1). The HRCT abnormalities were also stratified in relation to the “CT activity score” as already validated in previous studies [11,12] such that each lung was segmented into three zones as defined by Benamore et al. [11] and in each zone the extension of the following patterns was evaluated: ground-glass opacifications (GGOs), interlobular septa thickening (ITS), conglomeration, number of nodules. Finally, a sum of the scores for individual features was recorded (Table 2). For each term, we adopted the definition from the Fleischner Society glossary of terms [14] or by Akira et al. [15]. A “fibrotic lung” was defined when one of the following imaging features was present: honeycombing, traction bronchiectasis, reticulations, lobar volume loss.

All PFTs were performed according the ATS/ERS guidelines [16] at the Centre for Interstitial and Rare Lung Disease of our University Hospital, and included the following values: forced vital capacity (FVC), FVC%, forced expiratory volume (FEV), FEV1%, FEV1/FVC, diffusing capacity for carbon monoxide (DLCO)%. For each patient, the “Jaeger Vyntus Pneumo” manufactured by CareFusion (Carefusion, San Diego, CA, USA) was used. Six-minute walking tests were performed according to the ATS guidelines [17]. Patients’ personal data and medical history were collected through the analysis of the electronic medical records database. Patients were categorized in relation to respiratory patterns by a pulmonologist with proven experience in interstitial lung disease, blinded to imaging data. To comply with the exclusion criteria, a nephrologist (D.G.) re-evaluated the laboratory data and the doubtful cases were discussed collegially.

All patients provided their informed consent to archiving and processing of data for research purposes at the time of the examination. The images were archived in accordance with the Laws of the State and the internal regulations of our Institute.

### 2.3. Statistical Analysis

Data were collected on Microsoft Excel database (Microsoft Corporate, Redmond, WA, USA) and analyzed by MedCalc (MedCalc Software Ltd., Ostend, Belgium). Continuous variables were presented as means and standard deviations, and categorical variables were presented as percentages. The correlation between the CTAS and the variation of the respiratory function tests was evaluated with the Spearman’s ranked correlation test. The averages between groups of patients were assessed by the *t*-test for independent samples. Differences in outcome between two patient groups were assessed with the Fisher’s exact test. In our Box-and-Whisker plot, the central box represents the values from the lower to upper quartile (25 to 75 percentile), and the middle line represents the median; the upper limit of the orange box represents the arithmetic mean of the values.

The authors had full access to the data and take full responsibility for its integrity. All authors have read and agreed to the manuscript as written. For this retrospective analysis, it was not necessary to request authorization from our ethics committee. The contents of this paper are consistent with the principles of the Declaration of Helsinki in the latest version.

## 3. Results

### 3.1. General Population Characteristics

We critically reviewed the clinical history of 145 patients followed by the referral center for the diagnosis of sarcoidosis. Based on the eligibility and exclusion criteria, we enrolled a total of 55 patients (*N* = 55, Male = 35%). In our sample, the mean age was 59.2 (± 11.2; range 36–88 years old). At the time of diagnosis, the mean age was 52.2 (± 12.4), while the duration of illness in our sample were on average 6.78 years. The diagnosis of sarcoidosis was confirmed by histology in 30 (54%) cases, in 3 (5%) cases a Lofgren syndrome was assessed and finally the remaining 24 (43%) cases were classified by a positive BAL exam. The results have been summarized in Table 3. In the examined population, there was no clear correlation between the age at the time of diagnosis and the severity of the CTAS index (*r* = 0.01, *p* = 0.90), nor between the duration of the disease and the severity score referred to the first available control (*r* = 0.09, *p* = 0.48).

### 3.2. Relationship with Smoking and other Pollutants

**Figure 2:**
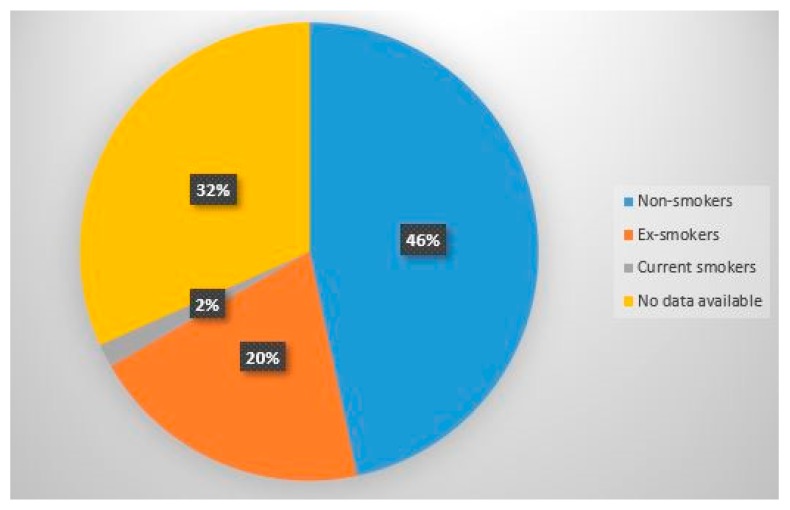
Proportion of patients exposed and not exposed to cigarette smoke.

The analysis of clinical documentation/records showed that in our series, 28 (50% of the total sample) patients never smoked, 12 (21% of the total) patients were ex-smokers for more than five years before the diagnosis of sarcoidosis, while only 1 (2% of the total) was a smoker; for 19 patients it was not possible to reconstruct with certainty the exposure to smoke, as it was not reported in the clinical documentation. Among smokers and ex-smokers, tobacco consumption has been estimated at an average of 16.3 packs per year (Figure 2).

**Figure 3:**
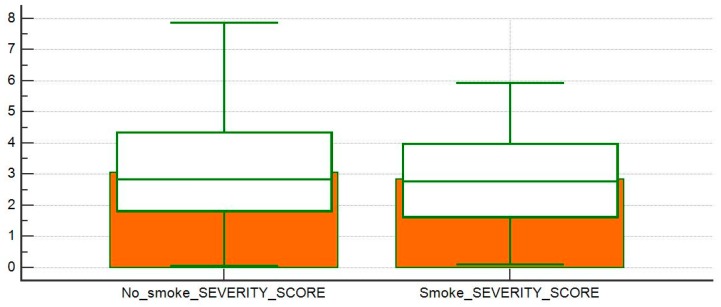
Comparison of severity score averages between exposed and non-smoking patients.

**Figure 4:**
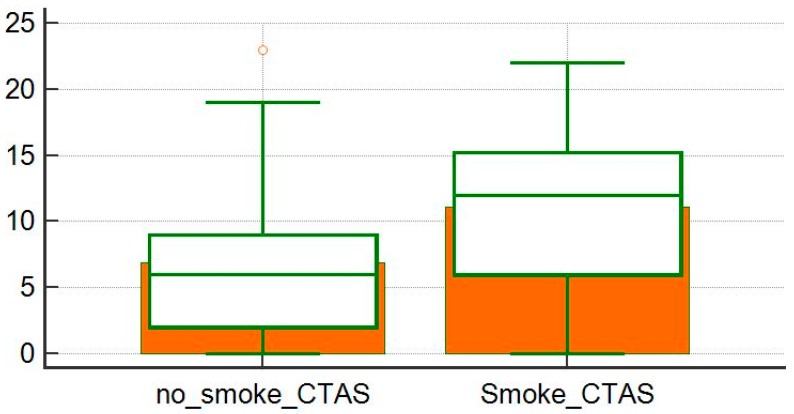
Comparison of computer tomography activity score (CTAS) averages between exposed and non-smoking patients.

Eleven patients (20%) had a documentable occupational exposure to dust, while five patients (9%) had a documentable environmental exposure to pollutants, organic dust, and mold. The examined population was divided into two groups: patients with a history of smoke exposure and patients without a history of smoke exposure. Among these two groups we compared means of the disease severity scores at the first visit and the CTASs related to the examined CTs. There was no statistically significant differences between the disease severity index applied to our samples (*p* = 0.72; Figure 3); on the other hand, the severity of HRCT manifestations, evaluated with the CTAS, was greater in the group with history of smoke exposure (*p* = 0.02; Figure 4).

### 3.3. Imaging Features

We identified that 29 (52%) patients showed atypical imaging characteristics (mean age 62.3 ± 11.3) while the remaining showed typical pattern (mean age 56.3 ± 10.6). A statistical difference was found comparing the two groups with *p* < 0.05 (confidence interval (CI): 95%).

**Figure 5:**
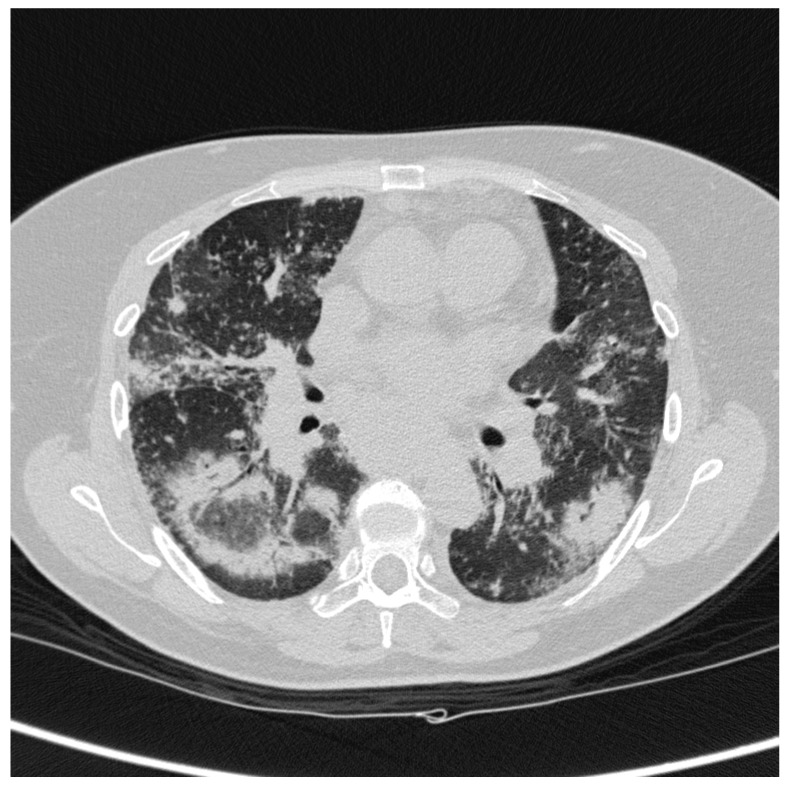
A patient affected by sarcoidosis (biopsy-proven). Axial scan in lung windows settings shows an area of ground-glass opacities (GGO) surrounded by denser consolidation of crescentic shape. This particular conformation is known as reverse halo sign or atoll sign.

In patients with atypical sarcoidosis (*n* = 29), the identified patterns sorted by frequency were as follows: nine patients (31% on atypical cases) with GGO, nine patients (31%) with atypical lymphadenopathy, eight patients (28%) with atypical nodules and masses (included a case of reverse halo sign; Figure 5), three patients (10%) had small airway involvement or air-trapping, three patients (10%) had atypical fibrotic alterations (asymmetric fibrosis, honey combing), three patients (10%) had emphysema, three patients (10%) had inter- and intra-lobular septa thickening, one patient (1%) had pleural effusion, and one patient (1%) showed atelectasis.

**Figure 6:**
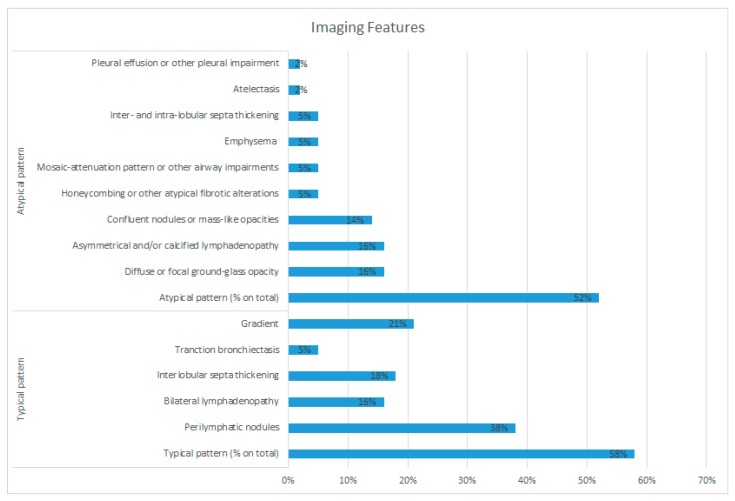
Proportion of imaging features.

In our series, 28% of patients had two or more features of atypical patterns such that one patient had fibrotic changes, GGOs and inter- and intralobular septa thickening; two patients had both lymphadenopathy and masses with atypical manifestations; one patient showed GGOs and pleural effusion; and one patient had atypical fibrotic changes, atypical lymphadenopathy, inter- and intra-lobular septa thickening, and atelectasis. These results are summarized in Table 3. In Figure 6 the relative proportion of the single features on atypical images are shown.

In the group of patients with atypical patterns, there was an inverse relationship between CTAS and DLCO%, however it was not statistically significant (*r* = −0.21, *p* = 0.3). No relationship was found between CTAS and FVC values (*r* = 0.02 *p* = 0.19) and FVC% (*r* = 0.06, *p* = 0.75). Similarly, in patients with typical patterns, no significant relationships between CTAS and FVC (*r* = 0.15, *p* = 0.43), FVC% (*r* = −0.17, *p* = 0.37), or DLCO% (*r* = −0.03, *p* = 0.9) were found.

### 3.4. Extra-Thoracic Localizations

**Figure 7:**
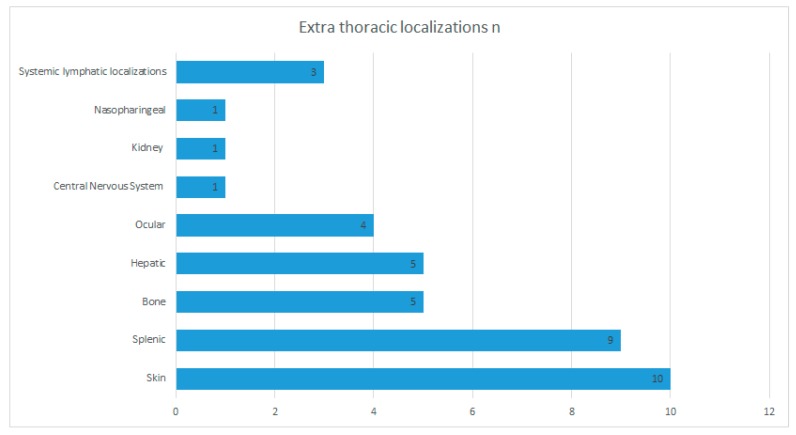
Proportion of extra-thoracic localizations.

In our series, the presence of extra-thoracic localizations of sarcoidosis was known in 27 patients (48% of our sample). Skin localization was the most frequent and was detected in 10 cases (37% of total extra-thoracic manifestations), followed by splenic (nine times, 33.3%), bones (five times, 18.5%), hepatic (five times, 18.5%), ocular (four times, 14.8%) involvement, and one localization only for central nervous system and renal and nasopharyngeal, respectively (each one accounting for 3.8%). Further systemic lymphatic localizations were recognized in three patients (Table 4 and Figure 7).

**Figure 8:**
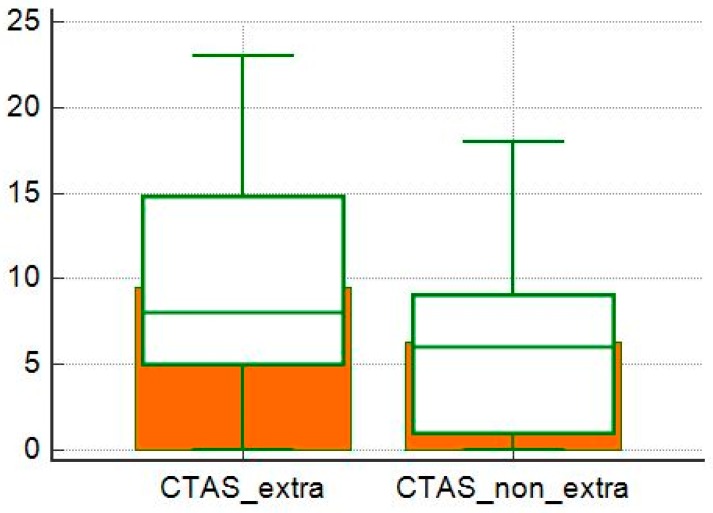
Comparison of CTAS averages between patient with extra-thoracic localization and without extra-thoracic localization.

**Figure 9:**
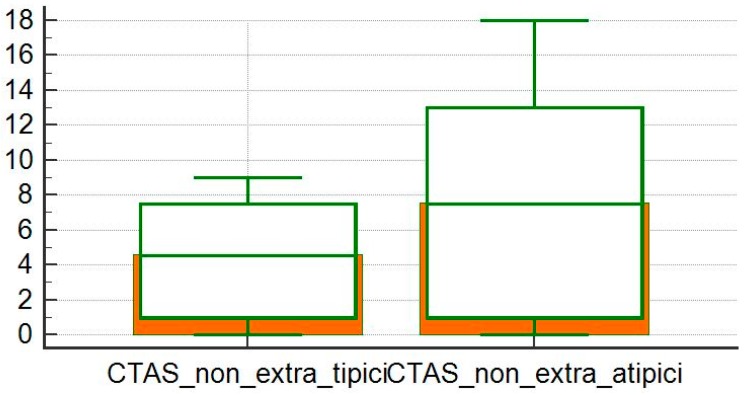
Comparison of CTAS averages between typical and atypical subgroups of patients without extra-thoracic localization.

**Figure 10:**
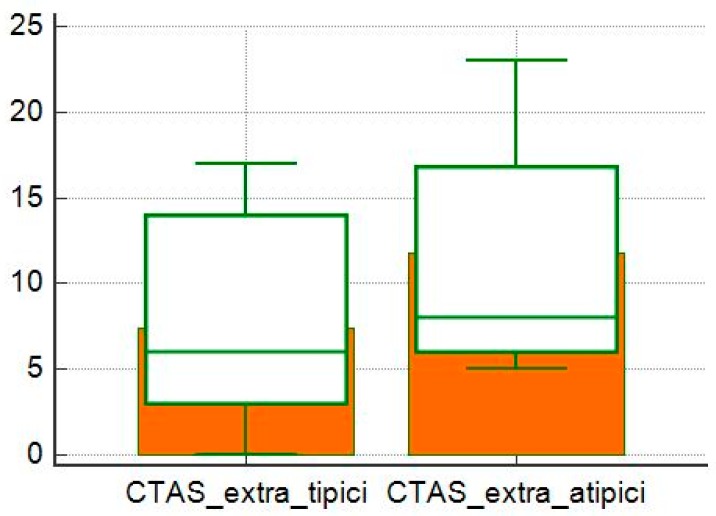
Comparison of CTAS averages between typical and atypical subgroups of patients with extra-thoracic localization.

The CTAS values were higher in patients with extra-thoracic locations than in patients with only intrathoracic locations (*p* = 0.05; Figure 8), but in these groups there was no difference between the severity of the HRCT between patients with typical and atypical patterns (*p* = 0.07 and *p* = 0.10; Figure 9 and Figure 10).

### 3.5. PFTs

**Figure 11:**
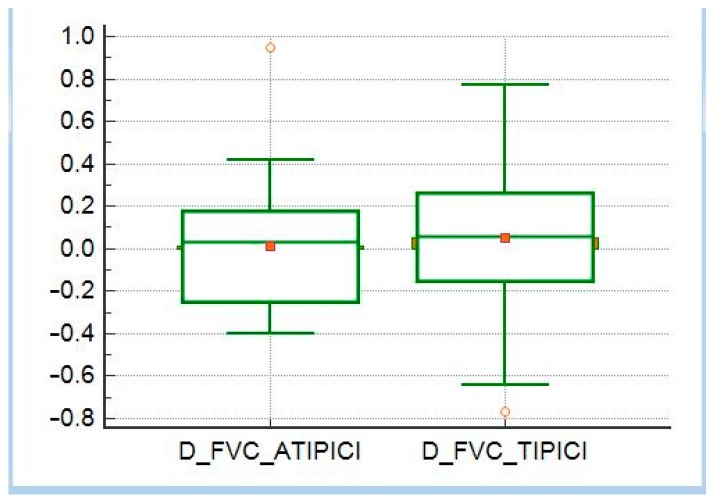
Comparison between means of diffusing capacity for carbon monoxide (DLCO) variation in patients with typical and atypical high-resolution computed tomography (HRCT) patterns.

**Figure 12:**
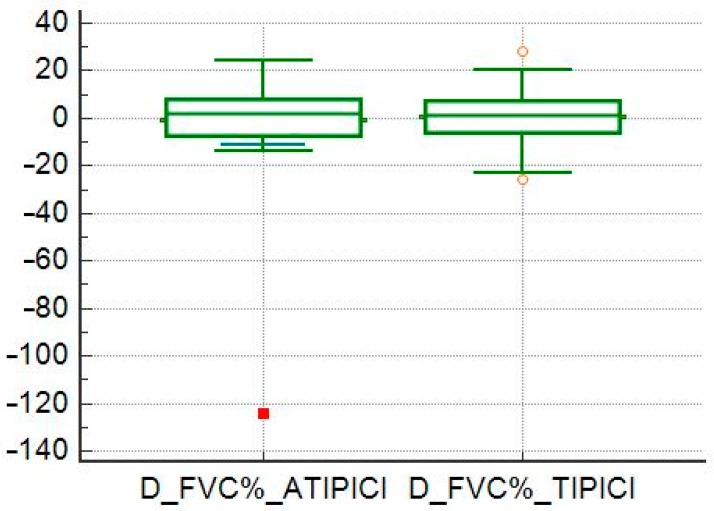
Comparison between means of forced vital capacity (FVC) variation in patients with typical and atypical HRCT patterns.

All patients included in this series completed at least two six-monthly respiratory function checks (PFTs); 73% of patients had at least three PFTs and 50% performed four PFTs. A six-minute walking test (6MWT) was available for 85% of patients and subsequent controls in 78% of cases. Through the analysis of PFTs, we found out that 28 patients (50% of the total) had a normal pattern of respiratory function, seven (13%) had restrictive patterns, 21 (38%) had obstructive patterns. Of the latter group, after reversibility testing, a positive response was observed in 18% of patients. We took into account the tests of respiratory function in two consecutive checks and we related this variation to the detected morphological patterns and also with the CTAS. In the whole sample, it was possible to determine that there was no correlation between CTAS and FVC (*r* = 0.02, *p* = 0.87), between CTAS and FVC% (*r* = −0.14, *p* = 0.32), while there was a weak correlation between CTAS and DLCO (*r* = −0.20, *p* = 0.01). In our analysis, we also compared the clinical outcome between patients with typical and atypical patterns, regardless of therapy. At one-year follow-up, there were no statistically significant differences between the two subgroups of patients in terms of variations of FVC and FVC% (*p* = 0.85 and *p* = 0.80; Figure 11 and Figure 12), while DLCO significantly worsens in patients with typical patterns compared to patients with atypical patterns (*p* = 0.00964). Finally, we evaluated the trend of the values (between two consecutive visits) of FVC, FVC%, and DLCO in the different subgroups. We found that there was an inverse correlation between the variation of FVC and the value of the CTAS (*r* = −0.3, *p* = 0.23) only in the atypical patterns subgroup. No further correlations were found.

### 3.6. Presentation Symptoms and Comorbidity

**Figure 13:**
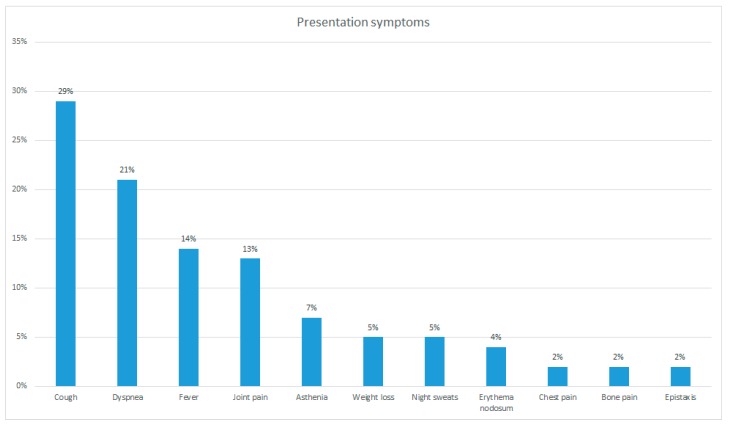
Prevalence of initial symptoms in the population studied.

The initial symptomatology in this sample was quite variable. Cough, dyspnea, and fever were the most frequent onset symptoms (in 29%, 21%, and 14% of cases, respectively). More rarely, the complained symptoms were joint pain (13%), asthenia (7%), weight loss (5%), and night sweats (5%). Chest pain, bone pain, epistaxis, and erythema nodosum were detected with a frequency of less than 3% (Table 5 and Figure 13). Some patients presented more than one symptom at the time of diagnosis.

**Figure 14:**
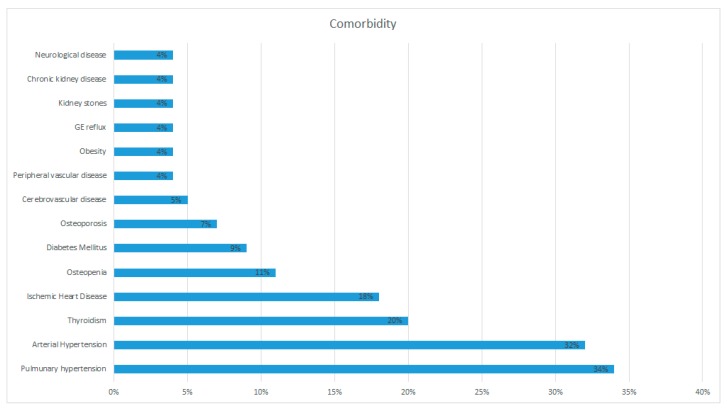
Prevalence of comorbidities in the studied population.

Several comorbidities were found in our series. Arterial hypertension was recognized in 18 patients (32%); ischemic heart disease in 10 patients (18%); thyroid conditions in 11 patients (20%); diabetes mellitus in five patients (9%); cerebrovascular disease in three patients (5%); and peripheral vascular disease, neurological diseases, gastro-esophageal reflux, kidney stones, chronic kidney disease and obesity in two patients (4%) each. Pulmonary hypertension was diagnosed in 19 patients (34% of the total), with a pulmonary artery pressure (PAPs) of 33.91 (±6.30). Osteopenia was found in six patients (12%), while four patients (7%) were affected by osteoporosis according to the WHO definition [18]. In accordance with the exclusion criteria, no patient of this sample was affected by an infectious, rheumatological, or active neoplastic disease (Table 6 and Figure 14).

The analysis of the available data did not allow us to highlight secure relationships between the initial symptoms or the comorbidity and the degree of severity of the disease or the manifestation in a specific pattern.

### 3.7. Pharmacological Treatment

In our retrospective analysis, 30 patients (54% of the entire sample examined) received drug treatment with glucocorticoids or immunosuppressants, while 25 patients (45.5%) did not need any therapy. In the first group, 12 patients had an atypical HRCT pattern and 18 had a typical HRCT pattern. At follow-up, there was a worsening of FVC and FVC% in 12 patients (of which five with atypical patterns) and of DLCO% in eight patients (of which three with atypical patterns). In the “non-therapy” group, 17 patients had an atypical HRCT pattern and eight had a typical HRCT pattern. At follow-up there was a worsening of FVC in 11 patients (eight with atypical patterns), a worsening of FVC% in 10 patients (seven with atypical patterns) and a worsening of DLCO% in 11 patients (nine with atypical patterns). Patients with atypical patterns most frequently needed therapy (*p* = 0.04). Regarding the worsening of FVC, FVC%, and DLCO%, there was no significant difference between the two groups (*p* = 0.77, *p* = 0.90, and *p* = 0.18). Subgroup analysis showed that there was no significant difference between the size of the group with extra-thoracic locations and the group with only thoracic locations (*p* = 0.89), but the subgroup of patients with extra-thoracic locations and atypical manifestations had a greater propensity in worsening in terms of variation of FVC (*p* = 0.03) and DLCO% (*p* = 0.04).

## 4. Discussion

Atypical patterns in sarcoidosis are found relatively frequently, even if its incidence seems to be variable in relation to different studies. In two previous Italian studies, which adopted similar diagnostic criteria, an incidence of atypical patterns was found between 30.3% [6] and 38.3% [9]. These data seem to be slightly lower than ours, in which patients with atypical patterns were 52%. This discrepancy could be related to the restrictive inclusion criteria adopted in our retrospective investigation. All these studies, however, are based on small populations, and this can contribute to data variability. As did other authors, we found that atypical patterns were a little more frequent in older patients, however in our series this tendency was not significant. Interestingly, it has not explained in literature why these patterns should be more frequent in older patients and the reason does not seem to be intuitive. Future meta-analyses on these series will be able to better evaluate the impact of age on the development of these features. Different HRCT patterns have a doubtful impact on the long-term outcome and response to therapy. In a retrospective study, Murdoch et al. have shown that patients with linear opacities have a greater propensity to worsening, while GGOs and other patterns were not useful for predicting the disease progression in a subsequent follow-up [19]. However, this study was limited by its small sample and the lack of clinical data for all patients. In contrast, another study by Akira et al. showed that GGOs and consolidations were associated with a worse prognosis, and patients with those findings were susceptible to developing severe respiratory insufficiency assessed with appropriate instrumental tests [15]. Polverosi et al. described a different response to therapy in typical and atypical patterns and demonstrated that immunosuppressant treatment could improve the clinical symptoms in both groups; in addition, immunosuppressors could reduce radiological findings in 50% of the typical cases, compared to 20% of atypical cases [6]. In these previous studies, however, the extent of thoracic lesions was not assessed with repeatable parameters or numerical scores. In order to characterize the extent of lung lesions, and to match imaging findings with clinical data, the analysis of radiological patterns was completed using a semi-quantitative score in our study. Analyzing the respiratory tests acquired at follow-up, we found that the worsening of DLCO values is related to CTAS such that in the atypical patterns group, there was an inverse correlation between FVC variations and CTAS values. Changes in lung function (assessed with DLCO values) seemed to be related to the degree of CTAS, and patients with typical patterns had a greater tendency to worsen than patients with atypical patterns. Based on these data, it is possible to state that, in our sample, both groups, at follow up, revealed a small but significant variation in at least one of the respiratory parameters, and the extent of this variation showed a slight correlation with the adopted visual score.

## 5. Conclusions

The use of reproducible scores, already validated in the literature, allowed us to compare imaging studies belonging to different patient subgroups. It has been found that patients with atypical patterns and patients with extra-pulmonary localizations (regardless of the HRCT pattern) have faster deterioration of respiratory function. Finally, there is a modest relationship between the CTAS values and the decline in respiratory function, probably in relation to a greater activity of the disease. An increased number of patients with atypical patterns will allow the investigation of the evolution of this heterogeneous pattern in the subgroups.

## Figures and Tables

**Table 1:** Criteria adopted for the definition of typical and atypical patterns.

Pattern	Features
Typical	
	Upper lobe predominance
	Nodules with perilymphatic and peribroncovascular distribution
	Mediastinal symmetric enlarged nodes
	Interlobular septa thickening, fibrotic alteration
	Traction bronchiectasis
Atypical	
	Diffuse or focal ground-glass opacity
	Confluent nodules
	Mass-like opacities
	Asymmetrical and/or calcified lymphadenopathy
	Honeycombing
	Emphysema
	Inter- and intra-lobular septa thickening
	Mosaic-attenuation pattern
	Pleural effusion or pleural

**Table 2:** Modified criteria adopted for the assignment of the computer tomography activity score (CTAS), from Duan [12].

Features	Partial Score
Ground-glass opacity (GGOs)	1%–25%: 1 point, 26%–50% 2 points, 51%–75% 3 points, and 76%–100% 4 points (for each zone)
Consolidation	1%–25%: 1 point, 26%–50% 2 points, 51%–75% 3 points, and 76%–100% 4 points (for each zone)
Inter-lobular septa thickening (IST)	Up to 5 was 1 point, over 5 was2 points (for each zone)
Nodules	1–25 was 1 point, 26–50 was 2 points, over 50 was 3 points (for each zone)
Conglomeration (over 2.5 cm)	No was 0 points, Yes was 1 point
Lynphoadenopaty	No was 0 points, Yes was 1 point

**Table 3:** Summary of imaging features.

Characteristics		*n* (% on Total Patients)	% on Total Patients	% on Atypical Patterns
General population characteristics	Total number of patients enrolled: 60			
	Age: 59.20 (± 11.24)			
	Sex (male; *n*, %): 25, 42%	25		
Diagnosis criteria				
	Histology	30	54%	
	Bronchoalveolar lavage (BAL ) +	24	43%	
	Loefgren syndrome	3	5%	
Pattern				
Typical		26	48%	-
	Age: 62.28 (±11.29)			
	Sex (male):	9		
Atypical		29	52%	-
	Age: 55 (±16.17)			
	Sex (male)	13		
Features				
	Diffuse or focal ground-glass opacity	10	17%	34%
	Asymmetrical and/or calcified lymphadenopathy	9	15%	31%
	Confluent nodules or mass-like opacities (included reverse halo sign)	8	13%	28%
	Honeycombing or other atypical fibrotic alteration	3	5%	10%
	Mosaic-attenuation pattern or others airway impairment	3	5%	10%
	Emphysema	3	5%	10%
	Inter- and intra-lobular septa thickening	2	3%	7%
	Atelectasis	1	2%	3%
	Pleural effusion or other pleural impairment	1	2%	3%

**Table 4:** Extra-thoracic localizations.

Organ or Tissue	*n*	%
Skin	10	37%
Splenic	9	33.3%
Bone	5	18.5%
Hepatic	5	18.5%
Ocular	4	14.8%
Central Nervous System	1	3.8%
Kidney	1	3.8%
Nasopharyngeal	1	3.8%
Systemic lymphatic localizations	3	11.1%

**Table 5:** Presentation symptoms.

Symptom	%
Cough	29%
Dyspnea	21%
Fever	14%
Joint pain	13%
Asthenia	7%
Weight loss	5%
Night sweats	5%
Erythema nodosum	4%
Chest pain	2%
Bone pain	2%
Epistaxis	2%

**Table 6:** Comorbidity.

Disease	*n*	%
Pulmonary hypertension	19	34%
Arterial Hypertension	18	32%
Thyroidism	11	20%
Ischemic Heart Disease	10	18%
Osteopenia	6	11%
Diabetes Mellitus	5	9%
Osteoporosis	4	7%
Cerebrovascular disease	3	5%
Peripheral vascular disease	2	4%
Obesity	2	4%
gastroesophageal (GE) reflux	2	4%
Kidney stones	2	4%
Chronic kidney disease	2	4%
Neurological disease	2	4%
